# DNMT3A mutations mediate the epigenetic reactivation of the leukemogenic factor MEIS1 in acute myeloid leukemia

**DOI:** 10.1038/onc.2015.359

**Published:** 2015-10-05

**Authors:** H J Ferreira, H Heyn, M Vizoso, C Moutinho, E Vidal, A Gomez, A Martínez-Cardús, L Simó-Riudalbas, S Moran, E Jost, M Esteller

**Affiliations:** 1 Cancer Epigenetics and Biology Program (PEBC), Bellvitge Biomedical Research Institute (IDIBELL), L'Hospitalet, Barcelona, Catalonia, Spain; 2 Department of Hematology, Oncology, Hemostaseology and Stem Cell Transplantation, Medical Faculty, RWTH Aachen University, Aachen, Germany; 3 Department of Physiological Sciences II, School of Medicine, University of Barcelona, Barcelona, Catalonia, Spain; 4 Institucio Catalana de Recerca i Estudis Avançats (ICREA), Barcelona, Catalonia, Spain

## Abstract

Close to half of *de novo* acute myeloid leukemia (AML) cases do not exhibit any cytogenetic aberrations. In this regard, distortion of the DNA methylation setting and the presence of mutations in epigenetic modifier genes can also be molecular drivers of the disease. In recent years, somatic missense mutations of the DNA methyltransferase 3A (DNMT3A) have been reported in ~20% of AML patients; however, no obvious critical downstream gene has been identified that could explain the role of DNMT3A in the natural history of AML. Herein, using whole-genome bisulfite sequencing and DNA methylation microarrays, we have identified a key gene undergoing promoter hypomethylation-associated transcriptional reactivation in DNMT3 mutant patients, the leukemogenic HOX cofactor MEIS1. Our results indicate that, in the absence of mixed lineage leukemia fusions, an alternative pathway for engaging an oncogenic MEIS1-dependent transcriptional program can be mediated by DNMT3A mutations.

## Introduction

Acute myeloid leukemia (AML) comprises a group of hematopoietic malignancies derived from myeloid precursors that have a highly heterogeneous clinical course and response to therapy. AML is characterized by greater proliferation and lower differentiation of the hematopoietic progenitor cells. Non-random cytogenetic aberrations are the single most important prognostic factor of the disease, but close to half of *de novo* AML cases do not exhibit any.^1^ Many molecular drivers with potential prognostic significance have been described particularly for this last group, such as mutations in nucleophosmin, fms-related tyrosine kinase 3 and CCAAT/enhancer-binding protein-α. In recent years, distortion of the DNA methylation setting and the presence of mutations in epigenetic modifier genes, such as Tet methylcytosine dioxygenase 2 and isocitrate dehydrogenase 1/2, have been directly implicated in the pathogenesis of AML.^2^ In this regard, somatic missense mutations of the DNA methyltransferase 3A (DNMT3A) have also been reported in ~20% of AML patients, in whom they are usually associated with an unfavorable prognosis.^3, 4, 5, 6^

DNMT3A is a *de novo* DNA methyltransferase that catalyzes the transfer of a methyl group onto the 5′-position of cytosine of CpG dinucleotides. Most of the DNMT3A mutations present in AMLs are heterozygous, with a great predominance of missense alterations in the R882 residue located in the catalytic domain.^3, 4, 5, 6^ R882H DNMT3A has recently been shown to act as a dominant negative that inhibits wild-type DNMT3A.^7^ In this context, AML samples carrying DNMT3A mutations have been found to be associated with DNA methylation changes.^8, 9^ However, no clear and common epigenetic signature has so far emerged and, most importantly, no obvious critical downstream gene has been identified that could explain the role of DNMT3A in the natural history of AML.

## Results and discussion

**Figure 1:**
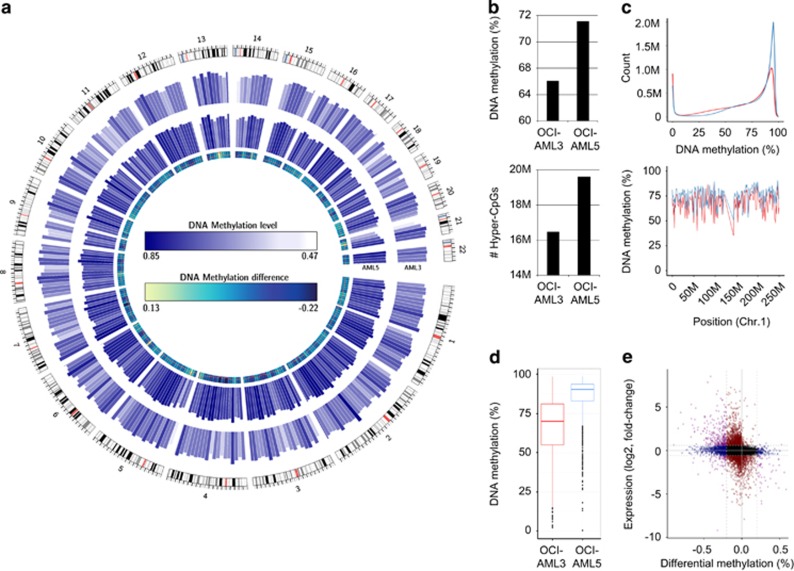
Complete DNA methylomes of DNMT3A wild-type and mutant AML cell lines. (**a**) Global DNA methylation levels in the DNMT3A mutant (OCI-AML3, outer circle) and wild-type (AML5, inner circle) cell lines analyzed by whole-genome bisulfite sequencing. Mean DNA methylation levels are displayed for 10 Mb genomic segments and all chromosomes. (**b**) Genome-wide analysis of DNA methylation levels at the CpG level (upper panel) and absolute number of hypermethylated (>0.66 methylation level; lower panel) CpG dinucleotides. (**c**) Genome-wide CpG methylation levels (upper panel) and DNA methylation profile exemplified by chromosome 1 (lower panel). (**d**) DNA methylation levels in DMRs hypomethylated in AML3. (**e**) Difference in promoter methylation (x axis; OCI-AML3 vs AML5) is associated with differential gene expression (y axis; OCI-AML3 vs AML5). Applied thresholds are indicated by dotted lines (δ DNA methylation >0.2; 1.5-fold change in gene expression). The 292 identified hypomethylated and overexpressed genes are highlighted in purple (upper left quadrant).

To find downstream hypomethylated targets mediated by the DNMT3A mutational event, we have taken an unbiased epigenetic approach to examine the entire DNA methylome at the single-nucleotide level of a well known DNMT3A AML mutant cell line (OCI-AML3, which harbors a heterozygous R882C mutation)^10^ and a widely used DNMT3A wild-type AML cell line (AML5). Using whole-genome bisulfite sequencing (Supplementary Methods), we generated 476 146 848 and 497 572 515 sequencing reads, of which 74.3% (353 777 108) and 80.4% (400 048 302) mapped uniquely to the human genome, respectively. Genome wide, we achieved a base coverage of 23.1x for OCI-AML3 and 26.1x for AML5 and 32.8x and 32.5x at CpG dinucleotides, respectively, enabling us to interrogate DNA methylation levels for >25 M CpG sites genome wide (>5 reads per site). The complete whole-genome bisulfite sequencing data from OCI-AML3 and AML5 are illustrated in Figure 1a, and are available for download from NCBI GEO (National Center for Biotechnology Information Gene Expression Omnibus): http://www.ncbi.nlm.nih.gov/geo/query/acc.cgi?token=crcvqguqdhwxrsf&acc=GSE62303.

We observed that DNMT3A mutant AML cells had a 9% (66.1% vs 75.1%) decrease in average DNA methylation level and fewer methylated CpG dinucleotides than did the DNMT3A wild-type cells (Figures 1b and c). The diminished methylated CpG dinucleotide content in OCI-AML3 observed with respect to AML5 cells is consistent with the reduced DNA methyltransferase activity associated with the mutations described in DNMT3A.^3, 4, 5, 6^ To find specific target genes affected by the DNA hypomethylation events noted in the AML cells harboring the DNMT3A mutation, we searched for particular differentially methylated regions (DMRs) between the two AML cell lines. These were defined as consecutively and consistently differentially methylated loci located beyond the 95% confidence interval (CI) of the smoothed methylation profiles. Using these criteria, we identified 182 800 DMRs between OCI-AML3 and AML5 cells. The most common DMR change was the presence of a methylated sequence in AML5 that was unmethylated in OCI-AML3: 156 919 hypomethylated events that represented 86% of the identified DMRs (Figure 1d). We focused on those hypomethylated DMRs located in unique candidate 5′-end regulatory promoters, which corresponded to a total of 1416 genes. To identify the hypomethylated promoters that had a transcriptional effect on the respective associated genes, we complemented the whole-genome bisulfite sequencing data with the results of an expression microarray experiment for the OCI-AML3 and AML5 cell lines (http://www.cancerrxgene.org/downloads/). This approach yielded 292 genes with transcriptional activation associated with promoter hypomethylation in DNMT3A mutant cells relative to wild-type cells (Figure 1e and Supplementary Table S1).

We next examined how the profile of genes with hypomethylation-associated expression derived from the DNMT3A AML cell line models translated to primary samples obtained from AML patients. To this end, we screened sixty-eight AML patients (whose clinical information is summarized in Supplementary Table S2) for DNMT3A mutations in exons 10–23 by direct Sanger sequencing; we also hybridized these samples to a comprehensive DNA methylation microarray that interrogates ~450 000 CpG sites. We detected 14 DNMT3A mutations (21%) in our AML group, a similar percentage to that reported previously,^3, 4, 5, 6^ consisting of 13 R882 mutations (7 R882H, 4 R882S and 2 R882P) and 1 S525C mutation. DNMT3A mutations were enriched in the AML cases that showed no cytogenetic abnormalities (Fisher's exact test, *P*=0.0353). None of our AML cases had mixed lineage leukemia (MLL) translocations. AML patients with DNMT3A mutations had a shorter 5-year overall survival (OS) (log-rank test; *P*=0.046; hazard ratio, 95% CI: 2.02, 1.00–4.10).

**Figure 2:**
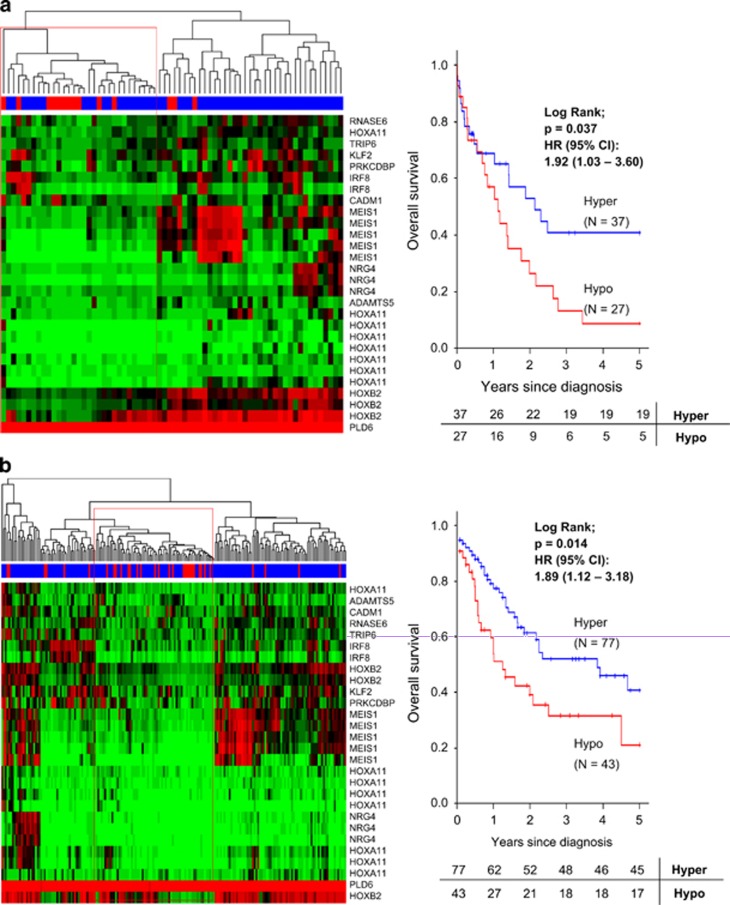
DNMT3A mutations in AML are associated with a DNA hypomethylation signature characterized by poor patient survival and MEIS1 induction. (**a**) Hierarchical clustered DNA methylation levels (green: 0% red: 100%) of 68 AML samples and 28 CpG sites significantly differentially methylated between DNMT3A mutant (red) and wild-type patients (blue). The red boxes indicate samples assigned to the DNMT3A mutant-related hypomethylated cluster. Differential survival analysis (5-year OS) of patients within (red line) or outside (blue line) the identified hypomethylated cluster (*n*=64, right panel). (**b**) Hierarchical cluster of the 28 CpG sites related to DNMT3A mutation in 194 primary AML patient samples.^2^ The red boxes indicate samples assigned to the DNMT3A mutant-related hypomethylated cluster. Differential survival analysis (5-year OS) of patients within (red line) or outside (blue line) the identified hypomethylated cluster in the independent patient cohort (*n*=139, right panel).

**Figure 3:**
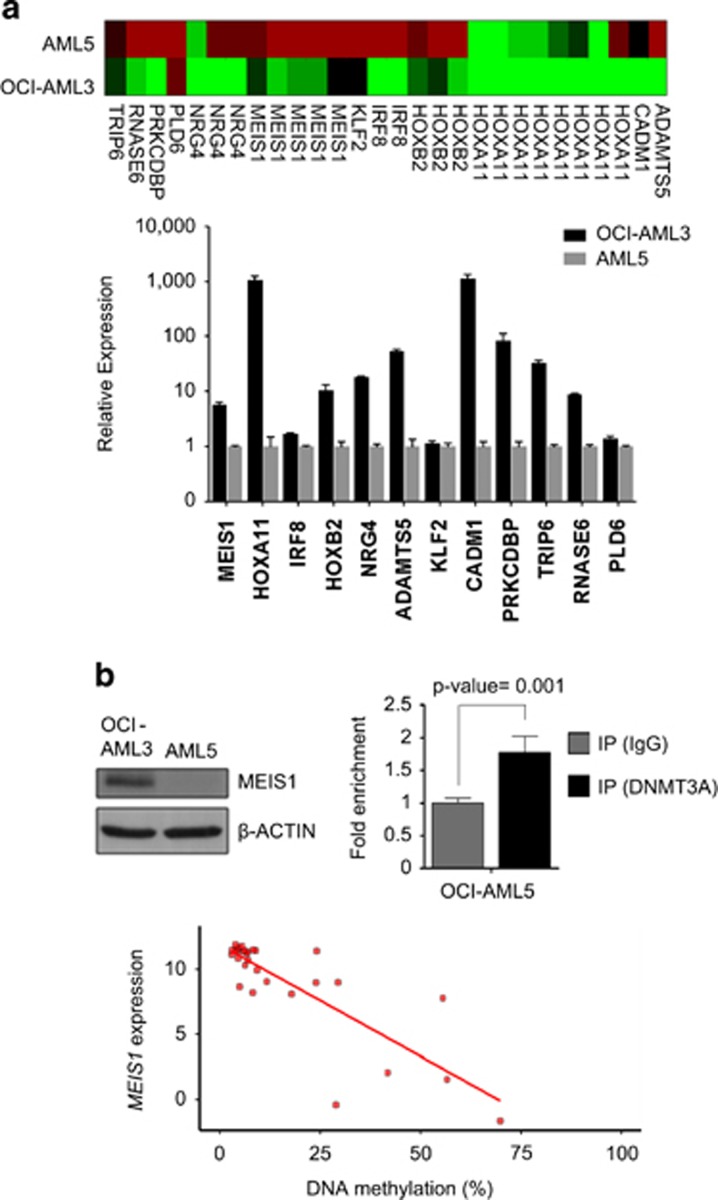
DNMT3A mutations in AML are associated with a DNA hypomethylation signature characterized by MEIS1 induction. (**a**) DNA methylation level of the 28 CpG sites among the 12 candidate genes in the OCI-AML3 and AML5 cell lines analyzed by DNA methylation array (upper panel). Relative gene expression levels of the 12 genes related to DNMT3A mutation profiled by quantitative PCR in OCI-AML3 (black) and AML5 (gray) cell lines (lower panel). (**b**) Protein levels of MEIS1 in OCI-AML3 and AML5 cells analyzed by immunoblotting (top left panel). Quantitative chromatin immunoprecipitation assay to assess DNMT3A occupancy at the MEIS1 studied CpG sites in AML5 cells. Data are presented as fold enrichment±s.e.m. Data of four independent experiments are shown. Significance of Student's *t*-tests is shown. IgG, immunoglobulin G (top right panel). Standard deviations are indicated by error bars. Bottom panel, association between the DNMT3A mutation-related differentially methylated CpG sites in MEIS1 and transcriptional activity using matched data from 170 primary AML samples (Cancer Genome Atlas Research Network^2^). Mean DNA methylation levels over the five CpG sites of MEIS1 and gene expression levels in DNMT3A mutant patients are shown.

When we combined the DNMT3A mutational status data with the DNA methylation analysis of our 292 identified hypomethylated-activated genes, we were able to define a signature of 12 hypomethylated gene promoters that were significantly enriched in the primary AML cases carrying the DNMT3A mutations (Wilcoxon's test; *P*<0.01) (Figure 2a and Supplementary Table S3). Interestingly, this 12-gene hypomethylation signature was also associated with worse OS (log-rank test; *P*=0.037; HR, 95% CI: 1.92, 1.03–3.60) (Figure 2a). The DNA hypomethylation signature of the 12 genes was validated in an independent cohort of primary AML patient samples (*n*=194),^2^ in which the described hypomethylated CpG sites were enriched in DNMT3A mutant patient samples (Fisher's exact test, *P*<0.01; Figure 2b). The 12-gene hypomethylated signature was also associated with shorter OS in this validation group (log-rank test; *P*=0.014; HR, 95% CI: 1.89, 1.12–3.18) (Figure 2b). We further confirmed by quantitative reverse transcription–PCR that the hypomethylated status of these candidate genes in the DNMT3A mutant OCI-AML3 cells was associated with a high level of expression of the corresponding transcripts, whereas their methylated status in DNMT3A wild-type AML5 cells was linked to transcriptional repression (Figure 3a). These target genes include two homeobox genes (HOXA11 and HOXB2), members of a family of transcription factors involved in differentiation that, it has been suggested, are hypomethylated in DNMT3A mutant AML.^8, 9^

However, most importantly, the highest-ranked candidate gene, whose five significantly differentially methylated CpG sites spanned the largest region among the 12 genes (Supplementary Table S3), was the leukemogenic HOX cofactor MEIS1. The reactivation of the *MEIS* gene at the protein level in the DNMT3A mutant cell line context was also confirmed (Figure 3b). The targeting of of the DNMT3A protein to the *MEIS* gene was observed using the chromatin immunoprecipitation assay (Figure 3b). We also noted the impact of DNMT3A mutant-mediated MEIS1 hypomethylation in the context of the primary patient,^2^ whereby AML DNMT3A mutant patients were hypomethylated and had a higher level of expression of MEIS1 (Spearman's correlation; *ρ*=−0.71, *P*<0.01) (Figure 3b). MEIS1 is critical for the development of hematopoietic cells and has highly regulated transcriptional activity with high levels observed in hematopoietic stem cells and early progenitor cells, but downregulated expression in later stages of hematopoietic development.^11^ This latter pattern appears to be disrupted in leukemogenesis, as persistent overexpression of MEIS1 has been consistently observed in association with poor prognosis in acute leukemia patients.^12^ In addition, MEIS overexpression causes shorter latency and accelerated progression in different leukemogenic models.^13, 14^ Interestingly, the common translocations in AML that involve MLL drive the activation of MEIS1 that is essential for the initiation and maintenance of MLL-rearranged AML.^13^ In this regard, MEIS1 overexpression in murine bone marrow progenitor generates an AML with features in common with those driven by the MLL-fusion proteins.^14^

Our results suggest that, in the absence of MLL fusions, as in our cases, an alternative pathway for engaging a leukemogenic MEIS1-dependent transcriptional program can be mediated by DNMT3A mutations. Under these circumstances, those AML patients carrying the alteration in the DNA methyltransferase would undergo a hypomethylation event at the MEIS1 promoter that would lead to the overexpression of this key oncogene in leukemia.^15^
