# Erratum: DNMT3A mutations mediate the epigenetic reactivation of the leukemogenic factor MEIS1 in acute myeloid leukemia

**DOI:** 10.1038/onc.2017.57

**Published:** 2017-03-13

**Authors:** H J Ferreira, H Heyn, M Vizoso, C Moutinho, E Vidal, A Gomez, A Martínez-Cardús, L Simó-Riudalbas, S Moran, E Jost, M Esteller

**Keywords:** Cancer epigenetics, Mutation, Transferases, Acute myeloid leukaemia

**Correction to:** *Oncogene* (2016) **35**, 3079–3082; doi:10.1038/onc.2015.359; published online 5 October 2015

**Updated online 13 March 2017:** This article was originally published under a CC BY-NC-ND 4.0 license, but has now been made available under a CC BY 4.0 license. The pdf and html versions of the paper have been modified accordingly.

