# Supplementary material for: DNMT3A mutations mediate the epigenetic reactivation of the leukemogenic factor MEIS1 in acute myeloid leukemia
Source: Oncogene. 2015 Oct 5;35(23):3079–82. doi: 10.1038/onc.2015.359 (PMC4705435; doi:10.1038/onc.2015.359)
Supplement: Supplementary Information [file onc2015359x1.doc]

**Supplementary Methods**

**Samples description**

OCI-AML3 and AML5 cell lines were obtained from the Leibniz Institute DSMZ-German Collection of Microorganisms and Cell Cultures (Braunschweig, Germany) and cultured according to supplier's specifications at 37ºC in a humidified atmosphere with 5% (v/v) CO_2_. Genomic DNA and RNA were extracted by the Phenol:Chloroform: Isoamylalcohol methodology (Sigma) and by automated purification with the Maxwell® 16 LEV simplyRNA Kit (Promega, Madison, WI, USA) following the manufacturer's protocol, respectively. DNA from primary AML samples was provided by the Department of Hematology of the RWTH Aachen University (Aachen, Germany) after written consent according to the “Biobank” rules of the medical faculty and approval by the local ethics committee (Permit Number: EK206/09).

**Whole Genome Bisulfite Sequencing**

We spiked genomic DNA (1 or 2 μg) with unmethylated λ DNA (5 ng of λ DNA per μg of genomic DNA) (Promega). We sheared DNA by sonication to 50–500 bp with a Covaris E220 and selected 150- to 300-bp fragments using AMPure XP beads (Agencourt Bioscience Corp.). We constructed genomic DNA libraries using the TruSeq Sample Preparation kit (Illumina Inc.) following Illumina’s standard protocol. After adaptor ligation, we treated DNA with sodium bisulfite using the EpiTect Bisulfite kit (Qiagen) following the manufacturer's instructions for formalin-fixed and paraffin-embedded (FFPE) tissue samples. We performed two rounds of conversion to achieve >99% conversion. We enriched adaptor-ligated DNA through seven cycles of PCR using the PfuTurboCx Hotstart DNA polymerase (Stratagene). We monitored library quality using the Agilent 2100 BioAnalyzer (Agilent) and determined the concentration of viable sequencing fragments (molecules carrying adapters at both extremities) by quantitative PCR using the Library Quantification Kit from KAPA Biosystems. We performed paired-end DNA sequencing (two reads of 100 bp each) using the Illumina Hi-Seq 2000.

Sequencing quality was assessed using the Illumina Sequencing Analysis Viewer and FastQC software. We ensured the raw reads used in subsequent analyses were within the standard parameters set by the Illumina protocol. Positional quality along the reads was confirmed to be QC>30, and we excluded biases towards specific motifs or GC-enriched regions in the PCR amplification or hybridization. Sequence alignment and DNA methylation calling of WGBS reads were performed using Bismark V.0.7.4 software (Krueger *et al*, 2011). SAM/BAM and BED file handling was done using SAMtools, bedtools (Quinlan *et al*, 2010) and Tabix (Li *et al*., 2011). Statistical analysis and graphic representation was performed with R (http://www.R-project.org) and multicore and ggplot2 libraries. We smoothed the DNA methylation profiles using a previously described method for processing WGBS data (Hansen *et al*, 2012). Briefly, the method assumes that the DNA methylation profile is defined by a varying function of the genomic location that can be estimated with a local likelihood smoother. We used HG19 as the reference genome and retrieved genomic information from Biomart (Haider *et al*, 2009) and Gencode V.16. The TSS was considered to be the most upstream base of all the annotated transcript variants of the gene.

**Infinium HumanMethylation450 BeadChip**

All DNA samples were assessed for integrity, quantity and purity by electrophoresis in a 1.3% agarose gel, picogreen quantification, and nanodrop measurements. All samples were randomly distributed into 96-well plates. Bisulfite conversion of 500 ng of genomic DNA was done using the EZ DNA methylation kit (Zymo Research), following the manufacturer’s instructions. 200 ng of bisulfite-converted DNA were used for hybridization on the HumanMethylation450 BeadChip (Illumina). The HumanMethylation450 BeadChip data were processed using the Bioconductor minfi package. We performed the “Illumina” procedure, which corrects for background signal and normalizes it, taking the first array of the plate as a reference. The methylation level (β) for each of the 485,577 CpG sites was calculated as the ratio of methylated signal divided by the sum of methylated and unmethylated signals plus 100. After the normalization step, we removed probes related to X and Y chromosomes. All analyses were performed in human genome version 19 (HG19).

.

**Expression and chromatin immunoprecipitation (ChIP) analysis**

Total RNA was reverse transcribed with the oligo-dT ThermoScript RT-PCR system (Invitrogen, Life Technologies, USA), according to the manufacturer's instructions. cDNA was amplified by real-time PCR using SYBR (Applied Biosystems) green detection and PPIA and GAPDH were used as housekeeping genes for normalization. Protein lysates were obtained using Laemli Buffer 1X after washing the cells with cold PBS and the respective concentrations were determined using the Bio-Rad DC protein assay (Bio-Rad Laboratories, Hercules, CA). Subsequent to standard techniques of western blot, membranes were incubated with Anti-MEIS1 antibody (ab19867, ABCAM) and β-actin-HRP (A3854, SIGMA). ChIP analysis for DNMT3A was performed as previously described (Jacinto *et al*, 2009). Primers are available upon request.

**References**

Krueger, F. & Andrews, S.R. (2011) Bismark: a flexible aligner and methylation caller for Bisulfite-Seq applications. *Bioinformatics*, **27**, 1571–1572.

Quinlan, A.R. & Hall, I.M. (2010) BEDTools: a flexible suite of utilities for comparing genomic features. *Bioinformatics*, **26**, 841–842.

Li, H. (2011) Tabix: fast retrieval of sequence features from generic TAB-delimited files. *Bioinformatics*, **27**, 718–719.

Hansen, K.D., Langmead, B. & Irizarry, R.A. (2012) BSmooth: from whole genome bisulfite sequencing reads to differentially methylated regions. *Genome Biology*, **13**, R83.

Haider, S., Ballester, B., Smedley, D., Zhang, J., Rice, P. & Kasprzyk, A. (2009) BioMart Central Portal--unified access to biological data. *Nucleic Acids Research*, **37**, W23–27.

Jacinto, F.V., Ballestar, E. & Esteller, M. (2009) Impaired recruitment of the histone methyltransferase DOT1L contributes to the incomplete reactivation of tumor suppressor genes upon DNA demethylation. *Oncogene*, **28**, 4212-4224.

**Sequence of Quantitative RT-PCR Primers**

| **Oligo Name** | **Sequence** |
| --- | --- |
| Hs-MEIS1-F | GACAATTTCTGCCACCGGTAT |
| Hs-MEIS1-R | TGATCTCTGTTCCAAGAGGGC |
| Hs-HOXA11-F | AGCCTCCCTTCTTTTCTGCC |
| Hs-HOXA11-R | GGCTCAATGGCGTACTCTCT |
| Hs-IRF8-F | CACGCTGGCAAGCAAGATTA |
| Hs-IRF8-R | CGGTCCGTCACTTCCTCAAA |
| Hs-HOXB2-F | CAAGAAACCCAGCCAATCCG |
| Hs-HOXB2-R | CAGCTGCGTGTTGGTGTAAG |
| Hs-NRG4-F | TCAACCCTACTCTCTTGACCA |
| Hs-NRG4-R | AACGACTTGTGACTGGGACC |
| Hs-ADAMTS5-F | GCATCTAAGCCCTGGTCCAA |
| Hs-ADAMTS5-R | TCGTGGTAGGTCCAGCAAAC |
| Hs-KLF2-F | TTCGCATCTGAAGGCGCATC |
| Hs-KLF2-R | GAGAAGGCACGATCGCACAG |
| Hs-CADM1-F | TCTGCTGTTGCTCTTCTCCG |
| Hs-CADM1-R | GGTCTGCCTGTTGGGATTCA |
| Hs-PRKCDBP-F | AGCTCCACGTTCTGCTCTTC |
| Hs-PRKCDBP-R | GCTCTGGTGCCTTCTGGAAA |
| Hs-TRIP6-F | GCAGGAAGAGGAAGAGGAGG |
| Hs-TRIP6-R | ACACTGGCCAAAGTACTCCC |
| Hs-RNASE6-F | CAACAGCTTCTGAGCTTTGGAC |
| Hs-RNASE6-R | GCTTAGGCCAAGCATGAAGT |
| Hs-PLD6-F | CGACTACATGGCCCTCAACG |
| Hs-PLD6-R | GTTGTTCTGGATGGCTTGCG |
| Hs-GAPDH-F | TGCACCACCAACTGCTTAGC |
| Hs-GAPDH-R | GGCATGGACTGTGGTCATGAG |
| Hs-PPIA-F | ATGGTCAACCCCACCGTGT |
| Hs-PPIA-R | TCTGCTGTCTTTGGGACCTTG |
