# Supplementary material for: DNMT3A mutations mediate the epigenetic reactivation of the leukemogenic factor MEIS1 in acute myeloid leukemia
Source: Oncogene. 2015 Oct 5;35(23):3079–82. doi: 10.1038/onc.2015.359 (PMC4705435; doi:10.1038/onc.2015.359)
Supplement: Supplementary Table 1 [file onc2015359x2.pdf]

Table S1: List of hypomethylated differentially expressed genes (OCI-AML3 vs AML5).

	Gene Expression			DNA methylation
Gene symbol	OCI-AML3	AML5	Fold change (log2)	AML3-AML5
AARS	10.00	7.69	2.32	-0.30
ABCB4	5.82	3.14	2.67	-0.45
ACAP1	4.77	3.40	1.37	-0.48
ACTN1	8.73	7.41	1.32	-0.20
ADAMTS5	5.52	2.94	2.58	-0.36
ADCK4	4.52	3.80	0.72	-0.24
ADORA3	5.23	3.64	1.59	-0.30
ADRBK1	6.16	5.07	1.10	-0.34
AFF3	6.17	4.69	1.48	-0.47
ALDH2	6.86	3.06	3.80	-0.49
ALDH9A1	9.02	7.54	1.47	-0.21
AMN1	6.44	3.91	2.53	-0.84
ANKRD13D	6.85	5.01	1.84	-0.32
ANPEP	5.00	3.22	1.77	-0.23
ANXA2	7.89	5.32	2.57	-0.24
APOBR	6.44	4.97	1.47	-0.38
APOC1	7.64	4.23	3.41	-0.25
ARHGAP1	6.71	5.73	0.99	-0.20
ARHGAP22	3.86	2.76	1.10	-0.38
ARHGEF17	4.13	3.36	0.77	-0.30
ART3	6.19	2.95	3.24	-0.29
ASGR2	8.25	3.30	4.96	-0.21
ATF4	12.84	11.74	1.10	-0.24
ATF5	7.74	4.26	3.48	-0.37
ATM	7.24	5.22	2.02	-0.47
AZI2	8.57	6.95	1.62	-0.24
B3GNT2	8.93	6.96	1.97	-0.26
B3GNT8	4.41	2.93	1.47	-0.35
BAK1	4.88	3.57	1.32	-0.24
BCL2A1	5.71	2.69	3.03	-0.28
BEX1	11.94	3.42	8.52	-0.41
BHLHB9	4.38	3.71	0.67	-0.39
BMP2	8.97	3.15	5.82	-0.26
BRSK1	4.74	3.54	1.20	-0.23
BTF3L4	8.62	7.35	1.27	-0.30
C11orf48	3.80	3.14	0.65	-0.28
C19orf59	9.12	4.70	4.42	-0.23
C1orf162	9.43	4.29	5.14	-0.32
C22orf46	4.85	4.10	0.75	-0.21
C2orf69	8.43	7.82	0.62	-0.34
C9orf64	6.92	4.57	2.34	-0.23
C9orf85	8.48	6.26	2.22	-0.51
CABP4	5.42	3.54	1.88	-0.42
CACNA2D4	8.30	4.25	4.05	-0.41
CADM1	6.58	3.37	3.20	-0.41

CAMK1	5.55	3.03	2.53	-0.49
CANX	10.54	9.72	0.82	-0.25
CCDC28B	6.01	4.14	1.87	-0.23
CCR1	7.48	3.91	3.57	-0.39
CD1D	10.30	5.94	4.36	-0.21
CD226	4.44	2.79	1.65	-0.23
CD320	5.87	3.52	2.35	-0.58
CD70	8.24	7.02	1.23	-0.29
CDA	5.09	3.11	1.98	-0.37
CDKN1B	7.84	7.17	0.67	-0.27
CDKN2B	3.60	2.97	0.63	-0.90
CECR6	9.56	3.58	5.98	-0.28
CENPH	8.93	7.80	1.13	-0.21
CFD	10.97	10.34	0.63	-0.25
CLSTN2	4.47	3.26	1.21	-0.23
CNBP	10.71	10.02	0.69	-0.31
COL15A1	6.05	3.55	2.50	-0.29
COPZ2	7.46	5.96	1.49	-0.32
CPNE8	7.72	5.06	2.66	-0.22
CRCP	6.70	5.67	1.03	-0.24
CRIM1	3.75	3.00	0.75	-0.20
CRK	6.41	5.79	0.62	-0.21
CTSG	12.92	9.25	3.67	-0.66
CYB5R2	4.72	3.17	1.55	-0.67
DENND2D	7.41	5.05	2.36	-0.26
DHRS9	11.20	9.84	1.36	-0.42
DNAJC4	5.82	5.14	0.69	-0.21
DOK2	7.75	4.84	2.91	-0.65
DUSP12	9.47	7.93	1.54	-0.25
EDEM1	5.39	4.69	0.71	-0.20
EEF1A1	6.66	6.05	0.60	-0.22
EFEMP2	5.26	3.88	1.37	-0.46
EIF2A	11.09	10.46	0.62	-0.35
EIF3M	8.87	8.26	0.61	-0.32
ELANE	8.35	3.06	5.29	-0.67
EMR2	4.54	3.82	0.71	-0.20
F2RL3	4.58	3.82	0.76	-0.30
FASTK	6.20	5.51	0.70	-0.22
FCER1G	11.17	3.34	7.84	-0.45
FCRLB	4.44	3.06	1.37	-0.22
FEZ1	7.65	3.14	4.51	-0.31
FEZ2	9.74	8.08	1.65	-0.37
FSTL4	3.91	3.30	0.61	-0.25
FUT7	5.85	4.09	1.76	-0.40
GALM	6.69	3.30	3.39	-0.46
GGPS1	7.60	6.93	0.67	-0.35
GNG12	3.57	2.98	0.59	-0.21
GPSM3	5.99	4.28	1.71	-0.25
GRAMD4	5.89	5.11	0.78	-0.31
GRSF1	8.47	7.70	0.77	-0.26

GSTT1	4.94	4.13	0.81	-0.33
GYG2	4.67	3.33	1.34	-0.46
HADHA	9.03	8.36	0.68	-0.44
HADHB	8.66	7.98	0.68	-0.43
HAL	7.58	5.35	2.22	-0.51
HENMT1	7.85	3.50	4.35	-0.80
HERPUD1	10.28	7.14	3.14	-0.26
HIVEP3	5.63	4.81	0.82	-0.27
HNMT	7.75	3.18	4.57	-0.33
HOXA11	8.78	2.99	5.79	-0.25
HOXA13	8.33	3.07	5.26	-0.23
HOXB2	6.70	3.03	3.67	-0.31
HS3ST4	4.33	2.92	1.41	-0.41
HSD17B8	6.67	3.75	2.92	-0.37
HYLS1	7.80	5.82	1.98	-0.35
ICAM3	7.16	5.35	1.81	-0.39
IFI16	9.57	5.12	4.44	-0.21
IGFBP3	4.65	3.10	1.55	-0.25
IL11RA	4.48	3.65	0.83	-0.26
IL31RA	4.53	3.39	1.14	-0.24
INPP4A	6.04	5.31	0.73	-0.30
IRF8	11.32	9.28	2.04	-0.40
IRX5	8.89	6.99	1.90	-0.54
ITGB2	11.35	10.55	0.80	-0.23
ITPKC	4.31	3.62	0.68	-0.25
KCTD11	4.33	3.69	0.64	-0.48
KDM2A	6.64	5.82	0.82	-0.30
KIAA0226L	4.12	3.31	0.81	-0.27
KIAA0930	8.62	7.66	0.95	-0.32
KIF7	4.62	3.77	0.85	-0.35
KLF11	5.28	4.06	1.23	-0.35
KLF15	5.27	4.41	0.86	-0.41
KLF2	7.65	5.37	2.28	-0.25
LAMP5	9.68	6.35	3.33	-0.24
LAT2	9.73	8.69	1.04	-0.24
LDHC	6.58	3.02	3.56	-0.62
LLGL2	5.14	4.43	0.71	-0.42
LPCAT1	10.19	8.05	2.13	-0.29
LPHN2	4.68	3.03	1.65	-0.23
LPPR3	6.36	5.23	1.13	-0.39
LRRC58	9.50	8.83	0.67	-0.24
LTBP3	3.96	3.31	0.64	-0.26
LTBR	7.49	3.60	3.89	-0.48
LXN	5.04	4.11	0.93	-0.36
MACROD1	5.25	4.45	0.79	-0.31
MAD1L1	8.54	5.96	2.58	-0.21
MAP1LC3A	5.46	2.95	2.51	-0.49
MBNL1	8.80	8.16	0.64	-0.23
MCOLN2	6.09	3.57	2.52	-0.29
MDM4	7.48	6.35	1.13	-0.22

MEIS1	5.63	3.44	2.19	-0.43
METTL8	8.33	7.61	0.71	-0.29
MGA	6.61	5.87	0.73	-0.60
MKX	9.76	2.91	6.85	-0.32
MLST8	8.35	5.34	3.01	-0.20
MLXIPL	5.69	4.42	1.27	-0.32
MMP14	7.32	4.79	2.52	-0.32
MMP2	4.52	3.38	1.14	-0.39
MNDA	11.22	9.38	1.85	-0.21
MS4A3	11.55	9.76	1.79	-0.33
MSN	11.01	9.85	1.17	-0.50
MST1	5.21	4.05	1.16	-0.26
MX2	7.04	6.23	0.81	-0.34
NAA38	8.52	5.89	2.63	-0.25
NAP1L5	5.95	2.67	3.27	-0.26
NAPG	6.95	5.68	1.28	-0.20
NCF4	10.82	9.34	1.48	-0.24
NEFH	5.59	2.91	2.68	-0.23
NEK8	5.56	4.20	1.36	-0.21
NFKBIL1	7.04	4.40	2.65	-0.35
NLRP3	8.31	7.59	0.72	-0.23
NME3	8.66	7.40	1.26	-0.26
NOLC1	7.93	7.32	0.61	-0.28
NPY1R	5.06	3.29	1.77	-0.30
NR1H3	4.64	4.00	0.64	-0.33
NRG4	7.30	3.33	3.97	-0.23
NT5E	5.81	3.16	2.65	-0.58
NT5M	7.31	5.94	1.37	-0.31
NUDT13	4.97	4.21	0.76	-0.25
NUMB	5.69	4.96	0.73	-0.23
NXF3	6.92	3.25	3.67	-0.26
ONECUT2	9.00	4.99	4.01	-0.23
OSBPL11	7.73	5.94	1.79	-0.33
OSBPL5	5.11	3.63	1.48	-0.44
P4HB	10.38	8.78	1.60	-0.31
PCM1	7.41	6.00	1.41	-0.31
PDGFRL	4.18	3.40	0.78	-0.41
PGBD5	4.04	3.36	0.68	-0.20
PID1	6.41	3.63	2.78	-0.21
PIGU	7.56	6.20	1.36	-0.27
PIK3AP1	8.56	7.73	0.83	-0.24
PLD3	4.98	3.05	1.93	-0.23
PLD6	7.02	4.19	2.83	-0.20
PLEC	4.90	4.12	0.78	-0.46
PPAPDC3	4.51	3.36	1.15	-0.34
PPIL3	10.40	8.88	1.52	-0.25
PPP4R2	7.82	7.02	0.80	-0.22
PRKCDBP	6.50	3.46	3.03	-0.77
PROCA1	4.71	3.23	1.49	-0.22
PSD4	4.68	3.73	0.96	-0.23

PSMD12	8.68	8.04	0.64	-0.22
PTCD2	4.16	3.14	1.02	-0.26
PTGER3	4.11	3.15	0.96	-0.31
PTPN7	7.33	6.00	1.33	-0.22
RAB5A	7.27	6.50	0.77	-0.27
RAC1	9.48	8.82	0.66	-0.26
RBBP9	6.74	5.22	1.52	-0.24
RBM38	7.76	6.47	1.29	-0.33
RBM47	5.65	3.40	2.25	-0.25
RFX7	5.15	4.50	0.65	-0.20
RINL	5.78	3.65	2.14	-0.37
RNASE6	10.46	3.98	6.48	-0.33
RNF114	7.96	7.25	0.71	-0.23
RNF157	4.31	3.59	0.71	-0.22
ROGDI	6.21	3.64	2.57	-0.21
RPGRIP1	3.76	3.14	0.62	-0.26
RPL36	7.04	6.39	0.65	-0.22
RPL39L	7.04	3.41	3.63	-0.20
RPL7L1	8.97	8.26	0.71	-0.25
RPS18	5.37	4.39	0.97	-0.37
RRAS	5.01	3.72	1.29	-0.23
RUSC1	5.24	4.30	0.94	-0.27
RXRA	7.61	6.73	0.88	-0.32
S100A11	10.36	7.21	3.15	-0.49
S100A13	5.45	4.03	1.41	-0.36
SAMHD1	7.77	4.61	3.16	-0.22
SCPEP1	8.62	7.48	1.14	-0.22
SEC23B	9.50	8.48	1.02	-0.29
SERPINH1	6.88	4.05	2.83	-0.23
SETDB1	6.76	6.13	0.63	-0.31
SFMBT2	6.28	3.74	2.54	-0.63
SGTB	6.39	4.47	1.92	-0.38
SIKE1	7.23	5.94	1.29	-0.25
SKA2	7.46	6.01	1.46	-0.29
SLC35D2	5.97	3.77	2.20	-0.62
SLC36A4	8.11	7.14	0.98	-0.23
SLC3A2	9.26	6.46	2.81	-0.24
SLC9A3	3.65	3.02	0.63	-0.20
SLFN12	4.74	2.64	2.10	-0.62
SOWAHC	7.16	3.28	3.88	-0.26
SP110	6.48	5.54	0.94	-0.23
SPAG9	5.63	4.86	0.77	-0.20
SPATC1L	5.41	4.62	0.79	-0.22
SPTLC2	9.01	7.12	1.89	-0.22
SRM	9.30	8.59	0.72	-0.20
STX12	7.20	6.44	0.77	-0.35
SUSD3	5.33	3.02	2.30	-0.46
SYAP1	6.96	6.06	0.91	-0.30
TAF6	4.12	3.52	0.59	-0.25
TARS	11.70	9.76	1.95	-0.21

TCIRG1	7.85	4.79	3.05	-0.30
TGFB1I1	4.72	3.89	0.83	-0.27
TLE3	6.36	4.71	1.65	-0.28
TLE6	4.79	3.25	1.54	-0.23
TMC6	4.66	3.72	0.94	-0.39
TMEM115	6.41	5.82	0.59	-0.32
TMEM128	7.50	6.38	1.12	-0.40
TMEM138	5.51	4.60	0.91	-0.26
TMEM167A	10.82	9.76	1.06	-0.32
TMEM209	7.32	5.79	1.52	-0.22
TMEM41B	6.43	5.51	0.92	-0.47
TMEM47	6.35	3.26	3.09	-0.33
TNF	7.28	3.35	3.93	-0.49
TOMM20	9.29	8.23	1.06	-0.28
TOX2	4.06	3.32	0.74	-0.22
TRIB2	8.37	3.34	5.03	-0.21
TRIP13	8.32	7.64	0.69	-0.22
TRIP6	9.17	4.29	4.87	-0.61
TRIT1	6.49	5.43	1.07	-0.20
TRPM2	5.79	4.47	1.32	-0.45
TSEN54	7.20	6.42	0.78	-0.25
TTC38	4.78	3.98	0.81	-0.26
TUBG2	6.27	4.48	1.79	-0.22
UBE2C	9.98	9.32	0.66	-0.33
UBE2M	9.27	8.44	0.83	-0.21
UBE2Z	7.37	6.48	0.89	-0.23
UGGT1	5.99	5.22	0.77	-0.27
UNC13D	4.92	3.88	1.04	-0.22
USP4	10.15	9.56	0.59	-0.26
VIT	7.23	3.34	3.89	-0.42
VKORC1L1	6.04	5.30	0.74	-0.24
VLDLR	7.71	4.12	3.59	-0.21
VPS4B	5.81	5.19	0.62	-0.21
WDR54	8.53	7.19	1.34	-0.24
WDR55	6.58	5.62	0.96	-0.27
XPOT	11.19	10.24	0.95	-0.21
YDJC	9.52	8.40	1.12	-0.29
YPEL3	7.14	4.08	3.06	-0.44
ZNF133	6.60	5.33	1.27	-0.22
ZNF331	6.98	4.92	2.06	-0.38
ZNF439	3.82	2.61	1.21	-0.41
ZNF451	5.67	4.99	0.68	-0.23
ZNF513	5.36	4.49	0.87	-0.22
ZNF532	5.47	3.65	1.82	-0.26
ZNF562	4.38	3.59	0.79	-0.24
ZNHIT1	9.57	8.85	0.72	-0.20
ZXDC	5.52	4.89	0.63	-0.23
