# Supplementary material for: DNMT3A mutations mediate the epigenetic reactivation of the leukemogenic factor MEIS1 in acute myeloid leukemia
Source: Oncogene. 2015 Oct 5;35(23):3079–82. doi: 10.1038/onc.2015.359 (PMC4705435; doi:10.1038/onc.2015.359)
Supplement: Supplementary Table 2 [file onc2015359x3.pdf]

Table S2. Clinical characteristics of the studied AML patient cohort

Characteristics	N	%	DNMT3A mutational status			
			Wild-type		Mutated	
			**N**	**%**	**N**	**%**
**Gender**						
Male	32	47.1%	26	81.3%	6	18.7%
Female	32	47.1%	24	75.0%	8	25.0%
Unknown	4	5.8%	4	100%	0	0%
**Age**						
< 50	19	28.0%	12	63.2%	7	36.8%
> 50	45	66.2%	38	84.4%	7	15.6%
Unknown	4	5.8%	4	100%	0	0%
**Risk group**						
Favorable	10	14.7%	10	100%	0	0%
Intermediate	39	57.3%	26	66.7%	13	33.3%
Adverse	8	11.8%	7	87.5%	1	12.5%
Unknown	11	16.2%	11	100%	0	0%
**Cytogenetics**						
Normal	31	45.6%	20	64.5%	11	35.5%
Complex	4	5.9%	3	75.0%	1	25.0%
Others	22	32.3%	20	90.9%	2	9.1%
Unknown	11	16.2%	11	100%	0	0%
**FAB subtype**						
M0 to M2	34	50.0%	27	79.4%	7	20.6%
M3 to M4	24	35.3%	19	79.2%	5	20.8%
M5 to M7	6	8.9%	4	66.7%	2	33.3%
Unknown	4	5.8%	4	100%	0	0%
**5-year Survival**						
Alive	24	35.3%	21	87.5%	3	12.5%
Exitus	40	58.9%	29	72.5%	11	27.5%
Unknown	4	5.8%	4	100%	0	0%
