# Supplementary material for: DNMT3A mutations mediate the epigenetic reactivation of the leukemogenic factor MEIS1 in acute myeloid leukemia
Source: Oncogene. 2015 Oct 5;35(23):3079–82. doi: 10.1038/onc.2015.359 (PMC4705435; doi:10.1038/onc.2015.359)
Supplement: Supplementary Table 3 [file onc2015359x4.pdf]

Table S3: Differentially methylated genes in 68 AML patients (Wilcoxon test, p<0.01).

**Gene symbol**	**DMR size (bp)**	**Expression fold change (log2)**	**CpG ID**	**# probes (450K)**	**Genomic location**
MEIS1	6926	2.19	cg05877497, cg22731271, cg26537478, cg01271812, cg08238215	5	chr2:66667059-66673985
HOXA11	3147	5.79	cg00418216, cg20994254, cg24446586, cg05311410, cg12997720, cg26857670, cg13352750, cg17950095	8	chr7:27224700-27227847
IRF8	186	2.04	cg16705546, cg04599946	2	chr16:85936480-85936666
HOXB2	90	3.67	cg01882880, cg21097733, cg17573933	3	chr17:46623729-46623819
NRG4	69	3.97	cg00438616, cg00371829, cg22211154	3	chr15:76304796-76304865
ADAMTS5	1	2.58	cg02462195	1	chr21:28340304
KLF2	1	2.28	cg05906166	1	chr19:16437057
CADM1	1	3.20	cg10193817	1	chr11:115375226
PRKCDBP	1	3.03	cg16776065	1	chr11:6340486
TRIP6	1	4.87	cg21406967	1	chr7:100464553
RNASE6	1	6.48	cg23595621	1	chr14:21250201
PLD6	1	2.83	cg26043529	1	chr17:17106266
